# Clinician Assessed Rates of PTSD and Complex PTSD in a Medical‐Rehabilitation Sample of Active‐Duty Military Personnel in the Armed Forces of Ukraine

**DOI:** 10.1111/acps.70050

**Published:** 2025-11-20

**Authors:** Philip Hyland, Mark Shevlin, Thanos Karatzias, Kristina Bondjers, Anna Scherbakova, Oksana Sulaieva, Anastasiia Bibikova, Olexandr Dudin, Anton Savchenko, Kseniia Voznitsyna, Victor Dosenko, Dmytro Martsenkovskyi

**Affiliations:** ^1^ Department of Psychology Maynooth University Kildare Ireland; ^2^ School of Psychology Ulster University Coleraine UK; ^3^ School of Health & Social Care, Edinburgh Napier University Edinburgh Scotland, UK; ^4^ National Centre for Disaster Psychiatry, Uppsala University Uppsala Sweden; ^5^ Medical Laboratory CSD Kyiv Ukraine; ^6^ Kyiv Medical University Kyiv Ukraine; ^7^ Scientific and Medical Center “DOSLID” Kyiv Ukraine; ^8^ Department of General and Molecular Pathophysiology Bogomoletz Institute of Physiology; NAS of Ukraine Kyiv Ukraine; ^9^ State Institution Veteran Mental Health and Rehabilitation Center Forest Glade Ministry of Health of Ukraine Kyiv Ukraine; ^10^ Department of Psychiatry Bogomolets National Medical University Kyiv Ukraine; ^11^ SI Research Institute of Forensic Psychiatry of MH of Ukraine Kyiv Ukraine

**Keywords:** complex posttraumatic stress disorder, military, posttraumatic stress disorder, soldiers, Ukraine

## Abstract

**Introduction:**

Millions of people have served in the Armed Forces of Ukraine (AFU) since Russia's invasion in 2014, but there is currently no information about the prevalence of posttraumatic stress disorder (PTSD) in this population. The main purpose of this study was to estimate rates of ICD‐11 PTSD and Complex PTSD (CPTSD), and comorbidity with major depression, in a sample of active‐duty, combat‐exposed AFU military personnel.

**Methods:**

Clinical interviews were conducted with 590 soldiers recruited from military hospitals and rehabilitation centers in Ukraine. All were trauma‐exposed during military operations. PTSD and CPTSD were diagnosed using the International Trauma Interview, and a current episode of major depression was diagnosed using the Mini‐International Neuropsychiatric Interview.

**Results:**

Overall, 67.4% of soldiers were diagnosed with ICD‐11 PTSD or CPTSD, with 45.9% being diagnosed with PTSD and 21.5% with CPTSD. Additionally, 34.4% were diagnosed with major depression, and comorbidity with PTSD (45.0%) and CPTSD (51.2%) was high. Elevated rates of PTSD were observed for current smokers and those who were currently consuming alcohol, while elevated rates of CPTSD were observed for officers (versus enlisted soldiers) and those recruited from rehabilitation facilities (vs. general hospitals).

**Conclusion:**

Although not representative of the entire AFU population, these results imply that hundreds of thousands of soldiers (and veterans) in Ukraine are likely experiencing clinically significant posttraumatic distress related to their combat experiences. Results are discussed in the context of finding scalable approaches to addressing this mental health challenge.

## Summary

- Significant outcomes
  ○ Among active‐duty, combat‐exposed Ukrainian soldiers, 45.9% were diagnosed with PTSD and 21.5% with Complex PTSD.
  ○ 34.4% were diagnosed with major depression, and comorbidity with PTSD (45.0%) and Complex PTSD (51.2%) was high.
  ○ Officers were more likely to be diagnosed with Complex PTSD whereas enlisted soldiers were more likely to be diagnosed with PTSD.
- Limitations
  ○ The sample was not representative of the entire population of the Armed Forces of Ukraine, limiting the generalizability of the results.
  ○ Inter‐rater reliability was not assessed limiting the interpretability of the results.

## 1 Introduction

Russia launched a war against Ukraine in 2014, escalating it into a full‐scale invasion in 2022, and continuing as of October 2025. At the outset of the war in 2014, the Armed Forces of Ukraine included 130,000 active personnel; that number now stands at just under one million active personnel supported by over one million reservists, making it the largest military force in Europe [1]. On February 16, 2025, President Volodymyr Zelenskyy reported that since the 2022 invasion, 46,000 Ukrainian soldiers had died, over 390,000 had been wounded, and tens of thousands remained missing in action [2]. Despite this enormous human cost, there are currently no estimates of the prevalence of posttraumatic stress disorder (PTSD) among Ukrainian soldiers or veterans—an essential metric for the effective planning and delivery of mental health services.

Examining data from other military populations who were recently exposed to combat can help establish expectations for PTSD prevalence within the Armed Forces of Ukraine. A meta‐analysis of 33 studies including nearly five million United States veterans who served in Afghanistan or Iraq reported an average PTSD prevalence rate of 23.1% (interquartile range was 19.2% to 29.0%) [3]. Several studies have also examined the prevalence of PTSD and Complex PTSD (CPTSD), as these disorders are defined in the *ICD‐11* [4], in military populations. A systematic review of 16 studies found that PTSD rates ranged from 3.8% to 42.4%, and CPTSD rates ranged from 5.0% to 80.6% [5]. Although prevalence estimates vary widely across studies, combat exposure is consistently found to be associated with higher rates of PTSD [6]. Furthermore, military personnel diagnosed with PTSD frequently experience comorbid mental health disorders, particularly major depression, which is present in about half of cases [7].

## 2 Aims of the Study

The primary aim of this study was to determine the prevalence rates of ICD‐11 PTSD and CPTSD in a sample of active‐duty, combat‐exposed members of the Armed Forces of Ukraine, as well as the comorbidity of these disorders with major depression. The secondary aim was to determine if prevalence rates of ICD‐11 PTSD and CPTSD varied significantly across a range of demographic and military‐related variables.

## 3 Methods

### 3.1 Participants and Procedures

For this study, 590 active‐duty, combat‐exposed military personnel from the Armed Forces of Ukraine were recruited from three hospitals and rehabilitation centers located in Dnipro, Ternopil, and Kyiv between November 2023 and May 2024. These data were collected as part of an ongoing project called the *Ukrainian National Initiative for Trauma studY* (UNITY study) which aims to examine the physical and mental health impacts of Russia's invasion of Ukraine on Ukrainian soldiers and veterans. The military personnel were drawn from either a rehabilitation facility (*n* = 125, 21.2%) or a medical hospital (*n* = 465, 78.8%), and the main reasons for hospitalization were intense stress reactions (*n* = 349, 61.8%), blast injuries (*n* = 192, 32.5%), bullet or shrapnel wounds (*n* = 31, 5.2%), and bone fractures (*n* = 18, 3.0%).

Recruitment was carried out by trained staff of the medical and mental health rehabilitation centers who provided potential participants with detailed information about the study and the voluntary nature of participation. Potential participants were assured that non‐participation would not affect their access to care. Written informed consent was obtained by members of staff before any data were collected. Ethical approval was obtained from the Ethics Commission at the Scientific Medical Centre “DOSLID”, Kyiv, Ukraine (Protocol No. 2 dated August 14, 2023), and the study protocol was approved by local ethical committees of the participating military hospitals and rehabilitation centers.

### 3.2 Measures

ICD‐11 PTSD and CPTSD: The International Trauma Interview (ITI) [8] is a semi‐structured, clinician‐administered interview used to assess all diagnostic requirements for ICD‐11 PTSD and CPTSD. Assessments were made by psychiatrists and clinical psychologists, all of whom completed a training course in how to administer the ITI. Assessors first established that participants were trauma‐exposed during combat operations and elicited an index trauma event against which symptoms were assessed. All participants identified a combat‐related experience as their most distressing traumatic event, and all experienced their index event more than 1 month prior to assessment.

The ITI includes six questions measuring PTSD symptoms across the three clusters of re‐experiencing in the here and now (nightmares and intrusive memories/flashbacks), avoidance (internal and external avoidance), and sense of current threat (hypervigilance and hyperarousal). These problems were assessed over the past month, and a five‐point Likert scale was used to record the severity of the symptom determined by combining information about the frequency and distress of the symptom (0 = absent, 1 = mild/subthreshold, 2 = moderate/threshold, 3 = severe/markedly elevated, 4 = extreme/incapacitating). Functional impairment related to these symptoms was assessed by two questions asking about impairments in social functioning and occupational or other important areas of life. The internal reliability of the PTSD items in this sample was acceptable (*α* = 0.80).

The ITI includes another six questions measuring the “disturbances in self‐organization” (DSO) symptoms that distinguish CPTSD from PTSD. These reflect persistent and pervasive problems in emotion regulation (difficulty calming down when upset and emotional numbing), negative self‐concept (feeling like a failure and feeling worthless), and disturbed relationships (feeling distant from other people and difficulty staying close to others). These problems were assessed in terms of typical reactions and recorded on a five‐point Likert scale (0 = not at all, 1 = a little bit, 2 = moderately, 3 = very much, and 4 = extremely). Functional impairment related to these symptoms was assessed by two questions asking about impairments in social functioning and occupational or other important areas of life. The internal reliability of the DSO items in this sample was acceptable (*α* = 0.83).

Scores on the Likert scale of 2 or higher are required for symptom and impairment endorsement. A PTSD diagnosis requires at least one symptom to be present from each PTSD symptom cluster and evidence of functional impairment related to these symptoms. A CPTSD diagnosis requires at least one symptom to be present from each PTSD and DSO symptom cluster and evidence of functional impairment related to both sets of symptoms (n.b., a person can only receive a diagnosis of PTSD or CPTSD, not both).

Major depression: Module A of the Mini‐International Neuropsychiatric Interview (MINI) [9] was used to diagnose a current episode of major depression (as per the DSM‐5‐TR criteria). The depression module of the MINI includes nine questions assessing the different symptoms, as well as one question assessing functional impairment. All questions were based on a binary “No” or “Yes” response format. A diagnosis of major depression requires that five or more symptoms are present, with at least one reflecting low mood or lack of interest in pleasurable activities, as well as the presence of functional impairment. The internal reliability of the nine depression items in this sample was good (*α* = 0.89).

Covariates: Participants were asked about their age, their marital and parental status, if they had completed a university education. Participants were also asked if they were currently smoking (Yes/No), and if so, how many cigarettes they smoked a day. Participants were asked if they were currently consuming alcohol (Yes/No), and if so, how frequently they were drinking with response options of “Never,” “Very rarely,” “Rarely,” “Sometimes,” and “Often.” Participants were asked about the military rank/status with response options of “Enlisted Soldier” or “Officer.” They were also asked if they had ever been held captive by Russian forces (Yes/No), and if so, what the duration of their captivity was (measured in months).

### 3.3 Analytic Plan

Descriptive statistics were used to summarize symptom, symptom cluster, and overall diagnostic rates for ICD‐11 PTSD and CPTSD, as well as the comorbidity between these disorders and major depression. To test for mean age differences based on diagnostic status (0 = no diagnosis, 1 = PTSD, and 2 = CPTSD), a one‐way between‐groups analysis of variance (ANOVA) test was conducted with Tukey post hoc tests. The effect size is reported using eta squared (*η* ^*2*^), where values can range from 0 to 1 and values up to 0.05 indicate a “small” effect, values from 0.06 to 0.13 indicate a “medium” effect, and values 0.14 and higher indicate a “large” effect. The Pearson chi‐square (*χ* ^*2*^) test was used to test for diagnostic status differences based on marital status (0 = not married, 1 = married), parental status (0 = not a parent, 1 = parent), university education status (0 = do not have a university degree, 1 = have a university degree), current tobacco use (0 = no, 1 = yes), current alcohol consumption (0 = no, 1 = yes), recruitment site (0 = general hospital, 1 = rehabilitation facility), military status (0 = enlisted soldier, 1 = officer), and history of Russian captivity (0 = no, 1 = yes). Effect sizes are reported using Cramer's *V*, where values range from 0 to 1, and values up to 0.3 indicate a “small” effect, values from 0.3 to 0.5 indicate a “medium” effect, and values of 0.5 and higher indicate a “large” effect [10].

## 4 Results

### 4.1 Sample Descriptive Statistics

The mean age of participants was 34.93 years (SD = 9.17, range = 20–61), and the sample was almost exclusively male (*n* = 589, 99.8%). Just over half were married (*n* = 317, 53.7%), 48.3% (*n* = 285) had children, and approximately one‐third had a university education (*n* = 209, 35.4%). Most participants were current cigarette smokers (*n* = 405, 68.6%), with the mean number of cigarettes smoked per day being 17.70 (SD = 8.16, range = 2–60). Regarding alcohol consumption, 19.8% (*n* = 117) stated that they currently consumed alcohol, and when asked about levels of alcohol consumption, 80.2% (*n* = 473) said “Never,” 11.9% (*n* = 70) said “Very rarely,” 4.4% (*n* = 26) said “Rarely,” 2.7% (*n* = 16) said “Sometimes,” and 0.8% (*n* = 5) said “Often.” Most participants were enlisted soldiers (*n* = 524, 88.8%) with the remainder being officers (*n* = 66, 11.2%). A small proportion had been held captive by Russian forces (*n* = 33, 5.6%), with a mean duration in captivity of 10.89 months (SD = 8.52, range = 0.5–29.0).

### 4.2 Diagnostic Rates

Rates of symptom, symptom cluster, and overall diagnostic rates are presented in Table 1. This information, where relevant, is also separated by diagnostic status.

**TABLE 1:** Symptom, symptom cluster, and overall diagnostic rates, separated by diagnostic status where relevant.

	Total (*N* = 590)	None (*n* = 192)	PTSD (*n* = 271)	CPTSD (*n* = 127)
	%	%	%	%
Posttraumatic stress disorder symptoms				
Nightmares	46.7	38.0	93.4	98.4
Flashbacks	40.2	6.3	52.4	65.4
Avoidance of internal reminders	78.5	36.5	98.5	99.2
Avoidance of external reminders	46.8	15.1	55.0	77.2
Hypervigilance	69.2	35.4	81.9	92.9
Hyperarousal	69.0	37.5	79.3	94.5
PTSD symptom impairment—social	76.1	33.5	94.8	100
PTSD symptom impairment—work/other areas of life	67.4	30.9	82.3	90.6
Disturbances in self‐organization symptoms				
Difficulty calming down	53.4	24.5	56.1	91.3
Numb or emotionally shut down	34.7	15.6	27.7	59.1
Feel like a failure	14.7	3.6	1.8	59.1
Feel worthless	18.0	3.1	1.8	74.8
Feel cut‐off from others	43.6	18.2	38.4	92.9
Difficulty staying close to others	32.2	9.4	25.8	80.3
DSO symptom impairment—social	53.2	20.3	54.6	100
DSO symptom impairment—work/other areas of life	43.2	20.8	37.3	89.9
Cluster endorsements				
Re‐experiencing in the here and now	80.0	38.5	100	100
Avoidance of traumatic reminders	80.0	38.5	100	100
Sense of current threat	85.4	55.2	100	100
PTSD symptom impairment	81.4	42.9	100	100
Affective dysregulation	62.7	34.4	65.3	100
Negative self‐concept	24.9	5.2	3.7	100
Disturbed relationships	48.1	20.8	43.2	100
DSO symptom impairment	56.8	27.6	57.2	100
Diagnosis				
ICD‐11 PTSD	45.9	—	—	—
ICD‐11 Complex PTSD	21.5	—	—	—
Either diagnosis	67.4	—	—	—

At the symptom level, endorsements ranged from 14.7% (feeling like a failure) to 78.5% (avoidance of internal reminders of the trauma). At the symptom cluster level, approximately 80% of participants met diagnostic requirements for each PTSD symptom cluster. Fewer participants met diagnostic requirements for the DSO symptom clusters, most notably the negative self‐concept cluster (24.9%).

At the diagnostic level, 67.4% (*n* = 398) were diagnosed with either ICD‐11 PTSD or CPTSD. Specifically, 45.9% (*n* = 271) were diagnosed with ICD‐11 PTSD and 21.5% (*n* = 127) were diagnosed with ICD‐11 CPTSD.

Additionally, 34.4% (*n* = 203) were diagnosed with a current episode of major depression, and there was significant comorbidity with ICD‐11 PTSD and CPTSD (*χ* ^*2*^ (2) = 87.19, *p* < 0.001, *V* = 0.38) with 45.0% of those diagnosed with PTSD, and 51.2% of those diagnosed with CPTSD, having a comorbid major depression diagnosis.

### 4.3 Demographic and Military Variables and Diagnostic Status

The results of the ANOVA and *χ* ^2^ tests to determine associations between diagnostic status and the nine covariates are presented in Table 2. No statistically significant effects were found for age, marital status, parental status, having a university education, or having been held in captivity by Russian forces. Statistically significant effects were identified for smoking status, alcohol use status, recruitment site, and military status. Specifically, rates of PTSD were significantly *higher* than expected by chance among smokers and those who were currently consuming alcohol, and significantly *lower* than expected by chance among officers (versus enlisted soldiers). Rates of CPTSD were significantly *higher* than expected by chance among officers (versus enlisted soldiers), and those recruited from a rehabilitation center (versus a general hospital), and significantly *lower* among those who were currently consuming alcohol.

**TABLE 2:** Associations between demographic and military variables and ICD‐11 trauma diagnosis.

	None (*n* = 192)	PTSD (*n* = 271)	CPTSD (*n* = 127)	*χ* ^2^ (*p*)	*V*
	%	%	%		
Married	58.9	52.4	48.8	3.45 (0.178)	0.08
Have children	46.9	48.7	49.6	0.26 (0.878)	0.02
University education	40.1	32.8	33.9	2.77 (0.251)	0.07
Current smoker	63.5	**74.2** ^a^	64.6	**7.15 (0.028)**	0.11
Current alcohol user	17.7	**28.0** ^a^	**5.5** ^b^	**28.42 (< 0.001)**	0.22
Recruitment site (mental health center vs. hospital)	**0.0** ^b^	21.4	**52.8** ^a^	**127.42 (< 0.001)**	0.47
Military status (officer vs. soldier)	9.9	**7.7** ^b^	**39.4** ^a^	**14.57 (< 0.001)**	0.16
Held in Russian captivity	6.8	5.5	3.9	1.17 (0.558)	0.04

	Mean (SD)	Mean (SD)	Mean (SD)	*F* (*p*)	*η* ^2^
Age	34.60 (9.22)	34.90 (9.02)	35.48 (9.44)	0.35 (0.774)	0.00

*Note*: All tests have 2 degrees of freedom; statistically significant effects are in bold.

Abbreviations: *χ* ^2^ = Pearson chi‐square test; *η* ^2^ = eta squared; *F* = Analysis of variance test; *p* = statistical significance; SD = standard deviation.

^a^ Significantly more cases in this cell than expected by chance.

^b^ Significantly fewer cases in this cell than expected by chance.

## 5 Discussion

Russia's war on Ukraine has been going on for over a decade, and despite the millions of people who have served in the Armed Forces of Ukraine during this time, there are no indicative estimates of the rates of PTSD/CPTSD in this population. Given the acknowledged death toll, and the well‐documented ferocity of the conflict [1, 2], it is very likely that a substantial proportion of service members, and veterans, are experiencing clinically significant levels of posttraumatic distress. This study sought to provide some insight into the rates of PTSD and CPTSD among active‐duty, combat‐exposed personnel in the Armed Forces of Ukraine. Before discussing the key study findings, it is important to acknowledge that the non‐representative nature of the sample means that these findings cannot be generalized to the entire population of the Armed Forces of Ukraine. Nevertheless, because of the relatively large sample, and the use of clinical assessments to diagnose PTSD and CPTSD, these findings do provide important insights into the level of clinical need that is likely to exist among Ukrainian soldiers.

The major findings of the study were that (i) just over two‐thirds (67.4%) of the soldiers interviewed met diagnostic requirements for either ICD‐11 PTSD or CPTSD, (ii) PTSD cases were more than twice as common as CPTSD cases, (iii) about one in three (34.4%) soldiers met diagnostic requirements for major depression, (iv) about half of all soldiers diagnosed with PTSD and with CPTSD had a comorbid diagnosis of major depression, and (v) PTSD and CPTSD diagnostic status were associated with smoking status, alcohol use status, site of recruitment, and military status.

Officers were much more likely to be diagnosed with CPTSD than enlisted soldiers (with enlisted soldiers much more likely than officers to be diagnosed with PTSD), and this effect may be related to the phenomenon of moral injury [11]. Moral injury refers to psychological distress that occurs when an individual's ethical principles are undermined by what they have done, failed to do, directly experienced, or witnessed. In the military, officers are responsible for giving orders to enlisted soldiers whom they command, and these orders can lead to soldiers being seriously injured or killed. It is possible that some officers will blame themselves for these outcomes and experience intense feelings of guilt and shame, increasing the risk of developing the various DSO symptoms that distinguish CPTSD from PTSD. This is something future studies with members of the Armed Forces of Ukraine could explore in more detail.

Those with PTSD were more likely to be currently smoking and consuming alcohol. It is well documented that persons with PTSD smoke and drink at higher levels than those without PTSD [12, 13], so these findings are not too surprising. It should be noted that alcohol consumption is prohibited by law among active‐duty military personnel in the Armed Forces of Ukraine, which explains the low base rate of alcohol consumption in the sample (i.e., 19.8%). Among those who did report that they were currently drinking, the majority (16.5%) stated that they consumed alcohol only “very rarely” or “rarely.” Thus, the finding that nearly 30% of those with PTSD consumed alcohol is noteworthy, and this may act as a useful clinical guide for detecting soldiers at higher risk of PTSD. Conversely, the base rate of cigarette smoking in this sample was very high (~69%), and although significantly higher in those with PTSD (~74%), it is potential usefulness as a clinical indicator of PTSD is probably limited given the pervasiveness of smoking among soldiers.

Finally, soldiers who were recruited from a rehabilitation facility were more likely to be diagnosed with CPTSD (but not PTSD) than those soldiers recruited from a general medical hospital setting. This is arguably good news; CPTSD is a more impairing disorder than PTSD, so the finding that rates of CPTSD are higher among those recruited from a rehabilitation facility (where rehabilitation can include psychological/emotional rehabilitation) implies that soldiers experiencing more intense levels of psychological distress are more likely to be referred to facilities that can provide interventions for emotional stabilization and recovery.

The current findings should be considered in relation to several study limitations. First, as already noted the sample was not representative of the entire population of the Armed Forces of Ukraine so the generalizability of these results is limited. Second, assessments of PTSD, CPTSD, and major depression were based on clinical interviews which may have affected results. Clinical interviews often produce discrepant prevalence estimates from self‐report assessments [14], and it is possible some participants were hesitant to report certain experiences (e.g., negative self‐concept, feelings of low mood) to an interviewer they did not know, or withheld information from the interviewer for fear of negative consequences. Moreover, it is also possible the interviewer's judgment of the person's reported experience was less than perfectly accurate. Future work would benefit from using direct self‐report assessment methods. Additionally, inter‐rater reliability was not assessed limiting conclusions regarding the consistency of ratings across different assessors.

Despite these limitations, these findings are important because they provide initial evidence about the prevalence of PTSD, CPTSD, and comorbid depression among soldiers in the Armed Forces of Ukraine. If the current results are even somewhat representative of the wider population of (combat‐exposed) soldiers in Ukraine, it would mean that hundreds of thousands of people are experiencing clinically meaningful levels of posttraumatic distress due to their combat experiences. This would represent an enormous challenge to the mental healthcare system in Ukraine which is already under extreme pressure following the full‐scale invasion in 2022 [15]. Military psychologists will have a critical role to play but current estimates suggest there is only one psychologist for every 400–500 soldiers in the Armed Forces of Ukraine; a ratio that compares unfavorably with, for example, the Israeli Defence Forces where there is one psychologist for every 70–90 soldiers. It is imperative therefore to find low‐intensity, evidence‐based treatments that can be delivered at scale to address this challenge. One promising treatment is Written Exposure Therapy (WET) [16], a five‐session treatment where individuals, alone or in groups, write about a traumatic event for 30 min. Multiple studies with military samples have shown that WET produces large reductions in PTSD and depression symptoms, comparable to other “gold standard” treatments for PTSD like Prolonged Exposure and Cognitive Processing Therapy, but with substantially lower rates of drop out [17, 18, 19]. The speed, simplicity, acceptability, and potential for automatization of this treatment makes it an ideal approach to managing the level of clinical need in this population.

## Author Contributions

All authors made a substantial contribution to the development and writing of the manuscript. A.S., O.S., A.B., A.S., K.V., V.D., and D.M. led the data collection and curation. All authors developed the research objectives. P.H. and M.S. did the statistical analysis. P.H., M.S., T.K., and D.M. wrote the first draft. All authors reviewed and revised the first draft to produce the final manuscript.

## Funding

This research was funded by the Government of Taiwan, whose generous financial support made the study possible. The authors acknowledge and deeply appreciate the commitment of both the Government of Taiwan and No Labels NGO to advancing scientific knowledge and supporting trauma research in Ukraine.

## Ethics Statement

Ethical approval was obtained from the Ethics Commission at the Scientific Medical Center “DOSLID”, Kyiv, Ukraine (Protocol No. 2 dated August 14, 2023), and the study protocol was approved by local ethical committees of the participating military hospitals and rehabilitation centers.

## Conflicts of Interest

The authors declare no conflicts of interest.

## Data Availability

The data that support the findings of this study are available on request from the corresponding author. The data are not publicly available due to privacy or ethical restrictions.
